# Integrated Metabolomics and Transcriptome Analyses Unveil Pathways Involved in Sugar Content and Rind Color of Two Sugarcane Varieties

**DOI:** 10.3389/fpls.2022.921536

**Published:** 2022-06-16

**Authors:** Zhaonian Yuan, Fei Dong, Ziqin Pang, Nyumah Fallah, Yongmei Zhou, Zhi Li, Chaohua Hu

**Affiliations:** ^1^Key Laboratory of Sugarcane Biology and Genetic Breeding, Ministry of Agriculture, Fujian Agriculture and Forestry University, Fuzhou, China; ^2^College of Agricultural, Fujian Agriculture and Forestry University, Fuzhou, China; ^3^Province and Ministry Co-sponsored Collaborative Innovation Center of Sugar Industry, Nanning, China; ^4^College of Life Sciences, Fujian Agriculture and Forestry University, Fuzhou, China; ^5^Center for Genomics and Biotechnology, Fujian Agriculture and Forestry University, Fuzhou, China

**Keywords:** sugarcane, metabolome and transcriptome, flavonoids, sugar metabolism, WGCNA

## Abstract

Metabolic composition can have potential impact on several vital agronomic traits, and metabolomics, which represents the bioactive compounds in plant tissues, is widely considered as a powerful approach for linking phenotype–genotype interactions. However, metabolites related to cane traits such as sugar content, rind color, and texture differences in different sugarcane cultivars using metabolome integrated with transcriptome remain largely inconclusive. In this study, metabolome integrated with transcriptome analyses were performed to identify and quantify metabolites composition, and have better insight into the molecular mechanisms underpinning the different cane traits, namely, brix, rind color, and textures in the stems (S) and leaves (L) of sugarcane varieties FN41 and 165402. We also identified metabolites and associated genes in the phenylpropanoid and flavonoid biosynthesis pathways, starch and sucrose metabolism. A total of 512 metabolites from 11 classes, with the vast majority (122) belonging to flavonoids were identified. Moreover, the relatively high amount of D-fructose 6-p, D-glucose6-p and glucose1-p detected in FN41L may have been transported and distributed by source and sink of the cane, and a majority of them reached the stem of sugarcane FN41L, thereby promoting the high accumulation of sugar in FN41S. Observations also revealed that genes such as C4H, CHS, F3H, F3’H, DFR, and FG2 in phenylpropanoid and flavonoid biosynthesis pathways were the major factors impacting the rind color and contrasting texture of FN41 and 165204. Further analysis revealed that weighted gene co-expression network analysis (WGCNA) hub genes and six transcription factors, namely, Tify and NAC, MYB-related, C2C2-Dof, WRKY, and bHLH play a key role in phenylpropanoid biosynthesis, flavone and flavonol biosynthesis, starch and sucrose metabolism. Additionally, metabolites such as L-phenylalanine, tyrosine, sinapaldehyde, pinobanksin, kaempferin, and nictoflorin were the potential drivers of phenotypic differences. Our finding also demonstrated that genes and metabolites in the starch and sucrose metabolism had a significant effect on cane sugar content. Overall, this study provided valuable insight into the molecular mechanisms underpinning high sugar accumulation and rind color in sugarcane, which we believe is important for future sugarcane breeding programs and the selection of high biomass varieties.

## Introduction

Sugarcane (*Saccharum* spp.) is a perennial C4 Graminaceous plant mainly cultivated in tropical and subtropical areas. It is widely known as a main sugar and biofuel feedstock crop that accounts for about 80% of global sugar production and 40% of ethanol production (Zhang et al., 2018). A number of studies have shown that a large quantity of sucrose is stored in the sugarcane stem, accounting for about 650 mM per kg (Welbaum and Meinzer, 1990). Furthermore, it has been reported that the quality of cane cultivar is contingent upon various conditions such as rind hardness, sucrose percent in juice and purity. High sugar content is one of the main objectives of sugarcane breeding, and increasing sugar content is economically important for the development of the sugarcane industry (Thirugnanasambandam et al., 2017). Bearing in mind the increasing demand for sugar by the world growing population, it is essential to produce cultivars with high sugar content. Cane varieties are known to have a close association with sugar yield and its yield-related parameters, namely, brix, stalk diameter, stalk weight, stalk number, stalk height, and fiber (Mancini et al., 2012). The vast majority of sugar-related agronomic traits such as HR brix, sucrose percent, number of green leaves, leaf area and internode length have demonstrated a significant relationship with rind hardness (Babu et al., 2009). The mechanisms underpinning sucrose accumulation have been investigated at various levels including identifying and characterizing individual metabolites (Glassop et al., 2007; Lin et al., 2022), transcriptome (Aitken et al., 2006), and genes in the sucrose pathway and movement of sucrose within plants (Moore, 2005), localization of genes (Rae et al., 2005), genomic maps advancement and quantitative trait loci localization (Casu et al., 2007).

In general, plants have developed different mechanisms to adapt to changing environmental conditions, for instance, the development of foliar trichomes, glandular hairs and a wax layer, and the production of metabolites (Granados-Sánchez et al., 2008). Previous studies showed that secondary metabolites including flavonoids, terpenoids, phenolics, proanthocyanidins, carotenoids, etc., are antioxidant agents (Rao et al., 2019), they also inhibit and deters oviposition and feeding. These metabolites can also protect plants against predators and pathogens (Ikonen et al., 2001), impede insect growth, attract pollinators (Agati and Tattini, 2010), and act as allelopathic agents (Sarker and Oba, 2018). Moreover, metabolites play crucial roles in protecting plant against fungi, bacteria, and viruses (Sun et al., 2008), and protect against ultraviolet radiation and high light (Rao et al., 2019). They can, besides, have potential impacts on other aspects of plant growth, development, and nutritional quality that are important in sugarcane production as well as different species and differ among plants of the same species, between diverse plant tissues (e.g., new and mature leaves, root, stem, fruit, etc.) (Rao et al., 2021). Therefore, metabolites provide immense potential in molecular breeding program. To sum up, it is of essence to investigate metabolites and their fundamental regulatory mechanisms from a more macroscopic standpoint such as metabolome.

Many thanks to the development of a high-throughput metabolite identification tool for sugarcane (Schaker et al., 2017), and the identification and quantification of all metabolites in biological samples (Patti et al., 2012). For instance, the metabolomics tool was employed to compare and quantify metabolites and their antioxidant activities in young and mature leaves of 12 different sugarcane varieties. It was revealed that the mature leaves of sugarcane varieties Taitang172 and ROC22 contained a significant amount of flavonoid, and these varieties exhibited high antioxidant activities among the 12 sugarcane varieties (Rao et al., 2021). In related study, Chen et al. (2018) detected 68 metabolites belonging to 11 metabolite classes, which varied considerably among the different tissues of Tieguanyin Tea cultivar using untargeted metabolomics. Wijma et al. (2021) also identified a high quantity of biosynthesis of secondary metabolites, amino acid metabolism, xenobiotics biodegradation and metabolism in different tissues of sugarcane. In sugarcane plants, several studies have been conducted using metabolomic analysis to study different biological problems. For instance, a previous study identified co-expression and specific metabolites associated with metabolic pathways correlated with Brix and fiber content using metabolite profiling (Perlo et al., 2020). In another study, targeted metabolomics tool was also employed to quantify 16 phenolamide and 90 flavonoid metabolites in the seedlings of different rice tissues (Dong et al., 2015). However, most of these previous studies only focused on metabolomics tool to investigate metabolites in different tissues of the plant. Whereas the integration of metabolomics with transcriptomics to investigate the different bioactive compounds and different potential transcriptional regulations in sugarcane, which is essential in tracking the changes of metabolites and their corresponding regulatory genes within specific cane tissues remains largely inconclusive.

Recently, metabolomics integrated with transcriptomics has been widely used to investigate the metabolites and related genes involved in biological pathways such as color variation and quality formation in many plants. For example, a combined transcriptomic and metabolomic analyses were adopted to identify the carbohydrate and organic acid metabolism genes associated with brix in of two types of tomato fruits. The study revealed that L-malic acid, citric acid, and genes involved in CHO metabolism were significantly associated with sugar content in tomato fruits (Li et al., 2021). Moreover, integrated transcriptome and metabolome tools were used to establish a global map of metabolite accumulation and gene regulation during fruit development in wild and cultivated watermelons (Gong et al., 2021). The analysis of metabolite and transcriptome profiles during the storage of two peach cultivars revealed the molecular mechanisms underlying different fruit textures in peach (Wang et al., 2018). Metabolic and proteomic analyses were also employed to identify potential proteins and pathways involved in sugarcane resistance (Wang et al., 2020). A previous study identified seven candidate genes involved in anthocyanin biosynthesis by transcriptomic and metabolomic analyses in three sugarcane cultivars of different colors. These authors identified some candidate genes associated with anthocyanin biosynthesis using transcriptomic and metabolomic analyses. They also found key flavonoids and anthocyanins that caused color difference, and the key candidate genes that regulated these metabolites (Ni et al., 2021). However, metabolites related to cane traits such as sugar content, rind color, and texture differences in different sugarcane cultivars using metabolome integrated with transcriptome remain largely elusive.

In the present study, we employed integrated metabolomic and transcriptomic analyses to detect and quantify the composition of metabolites in two distinct sugarcane cultivars (165204 and FN41), and to beer understand their relationship with cane traits such as sugar content, rind color, and texture. This study also aimed at identifying metabolites and associated genes in the phenylpropanoid and flavonoid biosynthesis pathways, and starch and sucrose metabolism. The results from this study will offer new insights on sugarcane stem growth and sugar accumulation and provide a theoretical basis for further research such as the validation of gene function and the genetic improvement of sugarcane cultivars.

## Materials and Methods

### Plant Materials and Growth Condition

Two sugarcane cultivars (“165204” and “FN41”) were cultivated in a randomized field plot according to standard agricultural practices in a field at the Baisha Town, Fuzhou City, Fujian Province, China (E 119°14’, N 26°16’) in 2019. The region has a subtropical monsoon climate with an altitude of 123 m, an average annual temperature of 17–20° and an annual rainfall of 1,200–2,100 mm. The site was previously used for sugarcane monoculture cropping system using a conventional approach. The following basic soil properties were measured: OM = 28.73 g/kg, total nitrogen (TN) = 1.22 g/kg, total phosphorus (TP) = 0.71 g/kg, and total potassium (TK) = 9.19 g/kg, this environment is suitable for sugarcane growth. The sugarcane cv. 165204 cultivated contained a green rind with a brittle texture, while cv. FN41 consisted of a purple rind with a hard texture. The treatments included (i) sugarcane monoculture with 165204 and (ii) sugarcane monoculture with FN41. Two varieties of sugarcane were cultivated on March 7, 2019, after the soil was plowed (40 cm depth) using rotary tillage. Sugarcane monoculture was cultivated with a line spacing of 1.2 and a planting density of 85,000 buds/hm^2^. The experiment was set in a randomized block design with two treatments and three replicates constituting a total of six plots, with each covering an area of 144.0 m^2^ (24.0 × 6.0 m). All plots were fertilized with the traditional local fertilizer application of 250 kg/hm^2^ of urea, 100 kg/hm^2^ of K_2_O, and 450 kg/hm^2^ of calcium superphosphate per season. Forty and sixty percent of the total fertilizer application were applied at the seedling and elongation stages of sugarcane, respectively. Sugarcane agronomic traits were investigated at the sugarcane maturation stage on 2 January 2020. The fresh stems and leaves of the two sugarcane varieties were collected on the same day, specifically, the cane stems of the seventh (middle) node of sugarcane, and the first fully expanded leaf of sugarcane as the material. Three biological replicates were collected for each tissue (stems and leaves) in two sugarcane cultivars (165204 and FN41), and a total of 12 samples were collected. “FN41L” and “165204L” represent the leaf tissues, while “FN41S” and “165204S” represent the stem tissues of sugarcane varieties FN41 and 165204, respectively. All the flesh samples were washed with DEPC water and 75% ethanol, wrapped in tin foil and labeled, then immediately placed in liquid nitrogen and stored at –80°C until further analysis.

### Analysis of the Properties of Sugarcane

To measure the stalk diameter and height of the plants, 30 sugarcane plants were randomly selected from each bed and measured with a tape and Vernier caliper. Extech Portable Sucrose Brix Refractometer (Mid-State Instruments, San Luis Obispo, CA, United States) was used to determine sucrose content and calculated through using the formula: sucrose (%) = Brix (%) × 1.0825 – 7.703. To understand the brittleness and stiffness of the stems of two sugarcane cultivars, we determined the mechanical properties of sugarcane stems by the method of testing in tensile strength perpendicular to the grain of wood. The tensile strength was measured according to the standard GB/T14017-2009. The cane stems of FN41 and 165204 cut into dumbbell shape and were tested using the UTM4304X electronic universal testing machine with a jig adapted to the tensile strength of sugarcane (model: JDSB104B). Test operation steps: In brief, we applied tensile force at a uniform speed along the main stem of sugarcane through the jig of the testing machine in the direction of the main stem until the stem was destructed (Supplementary Figure 1), and the tensile strength of sugarcane was calculated by adopting Elastic modulus (E) = (F/S) × (dL/L)^–1^. F represents the tensile strength, S stands the cross-sectional area of the sugarcane, dL denotes the elongation of the sugarcane, while L represents the original length of the sugarcane. The elastic modulus can be regarded as an indicator of the ease of producing elastic deformation of a material. The larger the value, the greater the stiffness of the material; the smaller the value, the more brittle the material.

### Sample Preparation and Extraction for Metabolomic Analysis

Firstly, the plant samples were freeze-dried in a lyophilizer (Scientz-100F, Ningbo, China), then ground to powder using a grinding instrument (MM 400, Retsch) for 1.5 min. Next, 100 mg of the powder was weighed and dissolved in 1.2 ml of 70% methanol extraction solution. Then, the dissolved samples were placed in a refrigerator at 4°C overnight and vortexed six times to improve the extraction rate. After overnight incubation, the mixture was centrifuged at 10,000 *g* for 10 min and the supernatant was filtered with a microporous membrane (SCAA-104, 0.22 μm pore size; ANPEL, Shanghai, China). The samples were stored in a sample injection bottle for UPLC-MS/MS analysis. Finally, a quality-control sample (mix) was prepared by mixing an equal amount of all samples to monitor the stability of the analytical conditions for assay analysis.

### Ultra Performance Liquid Chromatography and ESI-Q TRAP-MS/MS Conditions

Metabolite profiling was performed using an UPLC-ESI-MS/MS system [UPLC (Ultra Performance Liquid Chromatography), Shim-pack UFLC SHIMADZU CBM30A system^1^; MS/MS (Tandem mass spectrometry), Applied Biosystems 6500 Q TRAP]. The analytical conditions were as follow, UPLC: column, Waters ACQUITY UPLC HSS T3 C18 (1.8 μm, 2.1 mm*100 mm). The mobile phase consisted of solvent A, pure water with 0.04% acetic acid, and solvent B, acetonitrile with 0.04% acetic acid. Sample measurements were performed with a gradient program with the starting conditions of 95% A, 5% B. Within 10 min, a linear gradient to 5% A, 95% B was programmed, and a composition of 5% A, 95% B was kept for 1 min. Subsequently, a composition of 95% A and 5.0% B were adjusted within 0.10 min and kept for 2.9 min. The column oven was set to 40°C and volume of 2 μl. The effluent was alternatively connected to an ESI-triple quadrupole-linear ion trap (Q TRAP)-MS.

Linear ion trap (LIT) and triple quadrupole (QQQ) scans were acquired on a triple quadrupole-linear ion trap mass spectrometer (Q TRAP), API 6500 Q TRAP UPLC/MS/MS System, equipped with an ESI Turbo Ion-Spray interface, operating in positive and negative ion mode and controlled by Analyst 1.6.3 software (AB Sciex). The ESI source operation parameters were as follow: ion source, turbo spray; source temperature 550°C; ion spray voltage (IS) 5500 V (positive ion mode)/-4500 V (negative ion mode); ion source gas I (GSI), gas II(GSII) and curtain gas (CUR) were set at 50, 60, and 30.0 psi with a high collision gas (CAD), respectively. Instrument tuning and mass calibration were performed with 10 and 100 μmol/L polypropylene glycol solutions in QQQ and LIT modes, respectively. QQQ scans were acquired as MRM experiments with collision gas (nitrogen) set to 5 psi. DP and CE for individual MRM transitions were done with further DP and CE optimization. A specific set of MRM transitions were monitored accordingly for each period according to the metabolites eluted (Fraga et al., 2010).

### Metabolite Quantification and Data Analysis

Qualitative analysis of metabolites was performed according to the secondary spectrum information based on the self-built Metware Database (MWDB) of Metware Biotechnology Co., Ltd. (Wuhan, China) and other public databases of metabolite information including MassBank^2^, KNAPSAcK^3^, HMDB^4^, and METLIN^5^ (Zhu et al., 2013). Metabolite quantification was carried out with data acquired in the multiple reaction monitoring (MRM) mode of QQQ mass spectrometry. Mass spectrometry data were then analyzed and quantified using Analyst software v1.6.3 and Multiquant Software v3.0.2.

The data of metabolites profiling were pre-processed using unit variance (UV) scaling before multivariate analysis. Principal component analysis (PCA) was executed using the prcomp function in R software (version 3.0.3). Pearson’s correlation coefficient between samples was calculated in R using the cor function. Hierarchical cluster analysis (HCA) was performed using R package pheatmap based on the Euclidean distance coefficient. Further, orthogonal signal correction and Partial Least Squares-Discriminant Analysis (OPLS-DA) were executed after log2 transformation and Mean Centering of raw data by the MetaboAnalyst package in R software. The differentially expressed metabolites were screened based on OPLS-DA analysis by the following criteria: (1) Metabolites with fold change ≥ 2 or fold change ≤ 0.5; (2) Based on the above, the metabolites with VIP (variable importance in project) ≥ 1 were selected. We conducted a combine analysis between the metabolome and transcriptome datasets, the mean of all biological replicates of differential metabolites in the metabolome data and the mean value of expression of differential transcripts in the transcriptome data were examined. Later, we transformed the log2 datasets using the ‘cor’ package from the R software^6^. The Pearson correlation (*r*) was then employed between metabolites and transcripts in phenylpropanoid and flavonoid biosynthesis pathway, followed by starch and sucrose metabolism pathway was represented by network diagrams, and the genes and metabolites were selected when *R*^2^ > 0.8 (Cho et al., 2016). Metabolome and transcriptome relationships were visualized using the Cytoscape software version 3.6.1 (Su et al., 2014).

### Transcriptome Sequencing and Data Analysis

Total RNA was extracted from sugarcane samples using TRIzol reagent (Invitrogen, CA, United States) according to the manufacturer’s instructions. The isolated RNA was further treated with RNase-Free DNase (Promega, Madison, WI, United States) to remove possible genomic DNA. Qubit 2.0 fluorometer (Life Technologies, Carlsbad, CA, United States) and Agilent Bioanalyzer 2100 (Agilent Technologies, Palo Alto, CA, United States) were used to estimate the concentration and purification of the RNA, and its quality was confirmed using 1% agarose gel electrophoresis. High-quality RNA was used for further library construction. Library construction, library clustering and high-throughput sequencing were carried out by adopting Metware Biotechnology Co., Ltd (Wuhan, China) with an Illumina HiSeq™ 2500 platform (Illumina Inc., San Diego, CA, United States). Subsequently, the clean reads were obtained by removing the adaptors, reads with N greater than 10%, and whose base number with low-quality bases (*Q* < 20) were greater than 50%. The error rate, Q20, Q30, and GC content of the clean data were recorded to evaluate the RNA-seq quality. The raw RNA-seq read data were deposited in the Short Read Archive^7^ and can be accessed using the BioProject ID: PRJNA805530.

The clean reads were mapped to the reference sugarcane genome sequence using HISAT2 v2.1.0. Novel genes and transcripts were also predicted using StringTie v1.3.3b (Pertea et al., 2016). Subsequently, the gene expression levels of the samples were estimated as fragments per kilobase of exon model per million mapped fragments (FPKM) using featureCounts v1.6.1 (Liao et al., 2014). The differentially expressed genes (DEGs) were identified using DESeq2 v1.22.2 with | log2Fold Change| ≥ 1 and false discovery rate (FDR) < 0.05. Gene Ontology (GO) and Kyoto Encyclopedia of Genes and Genomes (KEGG) pathway functional enrichment analyses were performed via the Gene Ontology Database and KEGG Database^8^, respectively (Ashburner et al., 2000; Kanehisa et al., 2004). Fisher’s exact test was used to select the significant GO categories and KEGG pathways with the threshold of FDR < 0.05. Besides, we also annotated gene functions based on the following databases: NR (NCBI non-redundant protein sequences), KOG (euKaryotic Orthologous Groups) (Koonin et al., 2004), COG (Clusters of Orthologous Groups of proteins) (Tatusov et al., 2000), Pfam (Protein family) (Finn et al., 2014), Trembl (Translated EMBL Nucleotide Sequence Data Library) and Swiss-Prot (a manually annotated and reviewed protein sequence database) (Apweiler et al., 2004) with the BLAST program (Evalue ≤ 1e-5) (Altschul et al., 1997). Transcription factors (TFs) among the DEGs were predicted using iTAK online program^9^. Alternative splicing (AS) events were detected using rMATS v4.0.2. The SNP (Single Nucleotide Polymorphism) and indel (Insertion-Deletion) variants were called using GATK v3.8 and then annotated using ANNOVAR^10^.

### Co-expression Analysis

We used R package WGCNA (Langfelder and Horvath, 2008) to construct the gene co-expression network, and the genes with average gene expression greater than 10 were selected. After filtering, we obtained a total of 15,652 genes to construct the module. Some parameters are as follows: the soft thresholding power of the correlation network was set at 20, the deepSplit value was 2, the minimum gene module size was equal to 100, and the modules whose distance was less than 0.15 were merged and the total of 20 modules were generated. Later, Pearson correlation analysis showed that the module was co-expressed with the abundance of 24 metabolites related to phenylpropanoid biosynthesis (ko00940), flavonoid biosynthesis (ko00941), flavone and flavonol biosynthesis (ko00944), and starch and sucrose metabolism (ko00500). Finally, Cytoscape 3.6.1 was used to visualize the core genes in the core co-expression module (Kohl et al., 2011).

### Quantitative RT-PCR Validation

The expression level of genes was validated using Quantitative RT-PCR (qRT-PCR) according to the instructions of TransStart^®^ Top Green qPCR SuperMix (Transgen Biotech, Beijing, China). A total of 26 genes were selected and verified using qRT-PCR. The Gene-specific primers for qRT-PCR were designed with NCBI primer-blast tool^11^ and listed in Supplementary Table 1. The RNA samples used for qRT-PCR analysis were aliquots of the samples used in the RNA-seq experiments. Each qPCR reaction was performed using three biological replicates and three technical replicates. The PCR reaction conditions were as follows: 95°; for 10 min followed by 40 cycles of 95° for 30 s and 60° for 1 min. Reactions were performed using an Applied Biosystems 7500 Real-Time PCR system. The actin gene was used as the internal reference gene for normalization of expression, and relative expression was calculated using the delta-delta Ct method (2^–△△*Ct*^ method).

## Results

### Phenotype and Quality Traits Description of “FN41” and “165204”

Sugar content and texture of sugarcane are some of the most important indexes of sugarcane quality and have some significant relationship. Sugarcane with harder texture tend to produce more and sweeter sugar content, which are ideal for sugarcane squeezing, thus significantly reducing the production cost of sucrose and providing huge economic benefits for the sugar industries. On the other hand, the color difference of sugarcane has certain ornamental and economic value in the sugarcane industry. The sugarcane variety “FN41” has hard texture and purple color, with a higher sugar content of 17.22%, and “165204” cultivar texture consisted of a crisp and green ring color and 12.85% of sugar content. These two cultivars are deemed excellent materials for studying the mechanism of sugar content, texture and color difference of sugarcane. The findings of this study provide new insights into the molecular mechanism underpinning the accumulation of high sugar, hardness and color difference.

**FIGURE 1:**
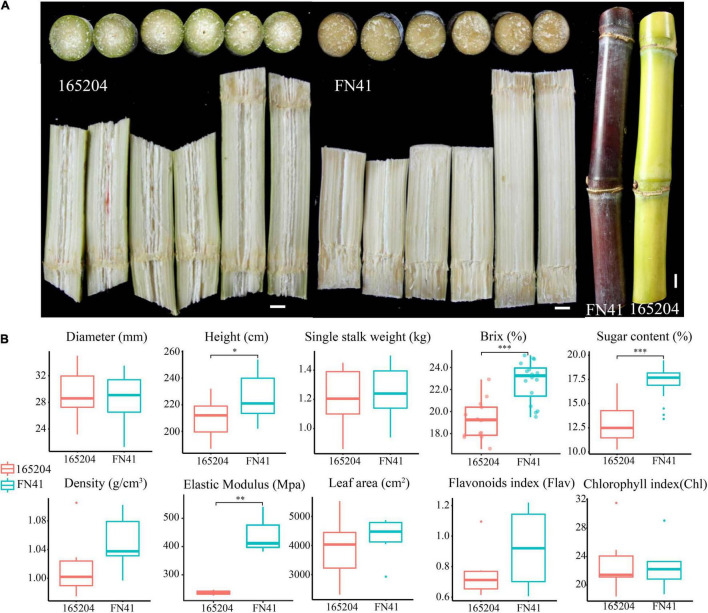
Physiological characteristics of FN41 and 165204 sugarcane cultivars, bar = 1 cm **(A)**; Comparison of agronomic traits between FN41 and 165204 **(B)**. *p < 0.05, **p < 0.01, and ***p < 0.001.

Although the two sugarcane cultivars were grown simultaneously in the same field and under the same conditions, the morphology of the tissues within the stalks and stem color were distinct (Figure 1A). FN41 had a purple rind, with fibers compressed fibers, while 165204 consisted of green rind and a loose connective fibrous tissue. During harvest, several important traits including the quality and yield of the two cultivars were evaluated (Figure 1B). Sugar content showed a significant differences were observed between FN41 and 165204 (percentage concentration of sugar, *p*-value = 2.80E-08) and stem height (*p*-value = 0.014). FN41 had a higher brix and stem height compared to 165204. While the other qualitative trait evaluated in our study were not significantly different between the two cultivars. Therefore, we speculated that the two cultivars of sugarcane have certain differences in sugar content, rind color, and pith texture.

### Metabolome Profiling and Identification of the Differentially Accumulated Metabolites Between FN41 and 165204

**FIGURE 2:**
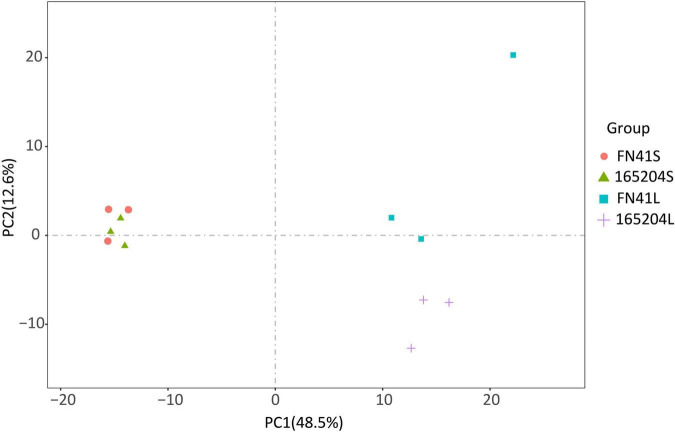
Principal component analysis (PCA) of the metabolites detected in the sugarcane stem and leaf samples with three biological replicates. “FN41L” and “165204L” represent the leaf tissues of sugarcane varieties FN41 and 165204, respectively; “FN41S” and “165204S” represent the stem tissues of sugarcane varieties FN41 and 165204, respectively, similarly hereinafter.

To quantify the total metabolites in the stems and leaves of the two varieties we adopted a metabolomics tool. A total of 512 metabolites grouped into 11 classes were identified from the 12 samples. Among them, there were 122 flavonoids, 89 phenolic acids, 67 amino acids and derivatives, 58 lipids, 44 organic acids, 35 nucleotides and derivatives, followed by 21 alkaloids, 7 lignans and coumarins, 3 tannins, 3 terpenoids, and 63 other metabolites (Supplementary Table 2). One QC sample was inserted for every 10 analyzed samples to monitor the reproducibility of the instrument’s analytical process. The overlay of the TIC plots between different quality control (QC) samples demonstrated the high repeatability and reliability of the data in this study (Supplementary Figure 2). We also performed PCA to visualize the metabolites composition in the samples. It was observed that metabolites composition in samples along PC1 (*x*-axis) accounted for 48.5% and PC2 (*y*-axis) represented 12.6% of variability, respectively. The analysis also revealed that metabolites composition in the stems of both varieties were densely clustered together, while metabolites composition in the leaf of both varieties leaf samples exhibited the opposite (Figure 2). The hierarchical cluster analysis (HCA) further revealed that the trends of metabolites composition were distinctly different between FN41 and 165204 (Supplementary Figure 3).

**FIGURE 3:**
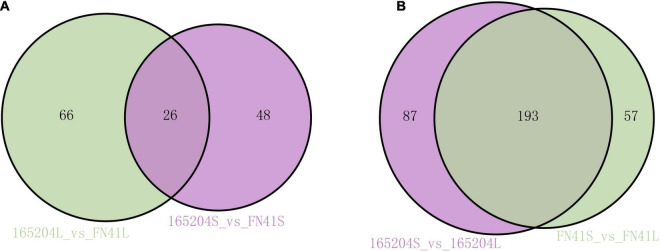
Venn diagrams illustrating differential metabolism between two varieties in the same compartments **(A)**, and in the stems and leaves of the same variety **(B)**.

**FIGURE 4:**
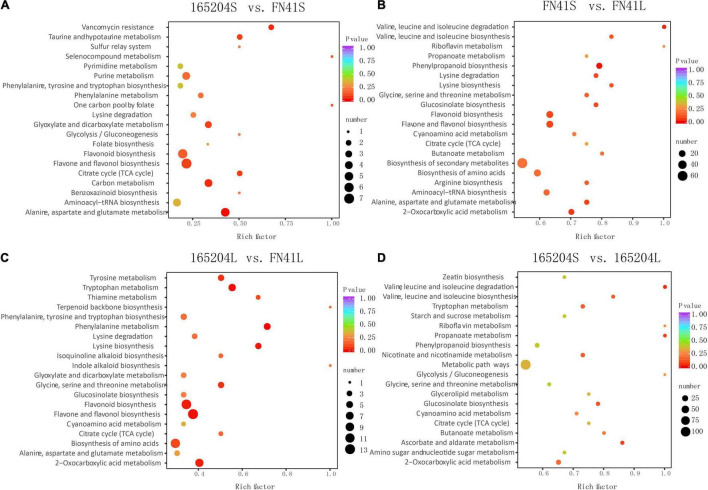
Kyoto Encyclopedia of Genes and Genomes enrichment analysis of the DEMs between **(A)** 165204S_vs_FN41S, **(B)** FN41S_vs_FN41L, **(C)** 165204L_vs_FN41L, and **(D)** 165204S_vs_165204L.

To pinpoint the significant differentially expressed dead-end metabolites (DEMs) associated with phenotype, the VIP (variable importance in project) ≥ 1.0 together with fold change ≥ 2 or ≤ 0.5 were set as the thresholds. We identified a total of 364 DEMs among the four groups compared (165204S_vs_FN41S, 165204L_vs_FN41L, 165204S_vs_165204L, and FN41S_vs_FN41L), including 74, 92, 280 and 250 DEMs in 165204S_vs_FN41S, 165204L_vs_FN41L, 165204S_vs_165204L, and FN41S_vs_FN41L, respectively (Supplementary Table 3), with most showing significantly high accumulation. In addition, only 26 DEMs were shared between the 165204L_vs_FN41L and 165204S_vs_FN41S, while 66 and 48 DEMs were exclusively associated with 165204L_vs_FN41L and 165204S_vs_FN41S, respectively. Nevertheless, the number of DEMs common in 165204S_vs_165204L and FN41S_vs_FN41L were 193, much larger than the 87 and 57 DEMs unique to 165204S_vs_165204L and FN41S_vs_FN41L (Figure 3). We also noticed that these DEMs were from distinct classes and mainly constituted flavonoids, phenolic acids, and amino acids and derivatives, suggesting that there were a variety of primary and secondary metabolites involved in different tissue and dissimilarity between species. KEGG pathway analysis among the DEMs revealed that KEGG pathways, including carbon metabolism, flavone and flavonol biosynthesis, flavonoid biosynthesis, phenylalanine metabolism, and phenylpropanoid biosynthesis were significantly enriched in the compared groups (Figure 4 and Supplementary Table 4). These results implied that the DEMs related to the flavone and flavonol biosynthesis, flavonoids biosynthesis, and phenylpropanoids biosynthesis are likely to play important roles in the different cultivars.

### Transcriptome Sequencing Revealed Differentially Expressed Genes in the Different Cultivars

**FIGURE 5:**
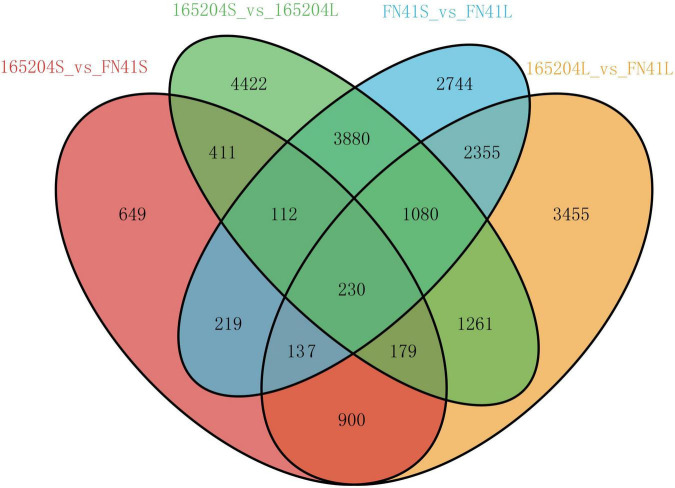
Venn diagram of the DEGs of the four comparison groups.

**FIGURE 6:**
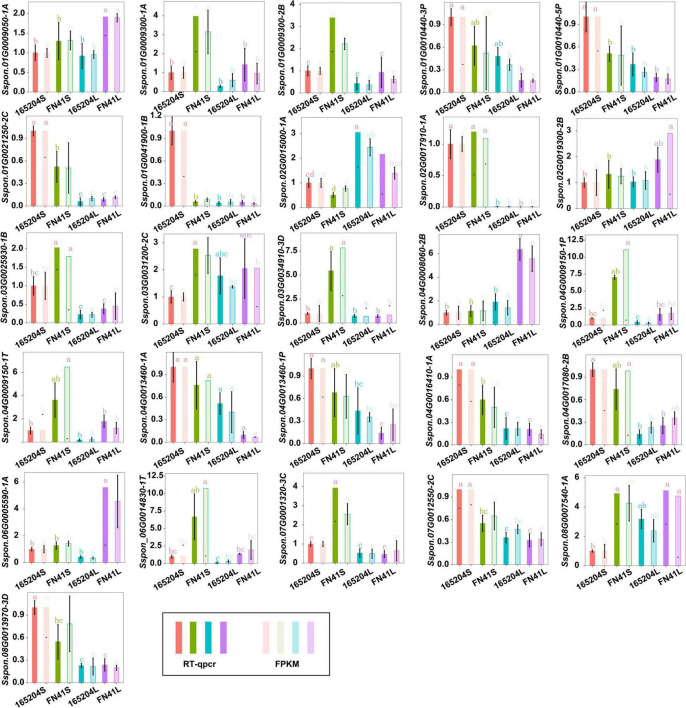
RT-qPCR analysis showing the significance level of various genes identified in the different tissues of cane.

To better understand the molecular basis of the metabolic differences detected in the different cultivars, transcriptome sequencing was performed using the stem and leaf tissues. A total of 104.52 Gb of clean reads was generated from the 12 libraries after removing the adaptor sequences and low-quality reads. The percentage of the high-quality score (Q30) was more than 93.92%, GC contents varied from 51.02 to 55.12%, and the successfully mapped ratio was more than 86.03% (Supplementary Table 5). The correlation coefficients between the biological replicates of the same tissues were greater than 0.88 (Supplementary Figure 4). These results indicated the high quality of the sequencing data. We carried out an evaluation of differentially expressed genes (DEGs) via the four pair-wise comparison groups (165204S_vs_FN41S, 165204L_vs_FN41L, 165204S_vs_165204L, and FN41S_vs_FN41L). The analysis revealed that 165204S_vs_165204L had the largest number of DEGs, consisting of 11,575, of which 6,628 were up-regulated and 4,947 were down-regulated (Figure 5). Whereas a lower number of DEGs was identified in 165204S_vs_FN41S, with 2,837, of which 1,938 were up-regulated and 899 were down-regulated. The comparison of FN41S_vs_FN41L resulted in 10,757 DEGs, including 6,419 up-regulated and 4,338 down-regulated. The comparison of 165204L_vs_FN41L revealed a total of 9,597 DEGs, among which 6,115 were up-regulated and 3,482 DEGs were down-regulated. The detailed information about the diversity of DEGs is available in Supplementary Table 6. The results of DEGs between different comparison groups indicated that the gene expression profiles varied significantly between these two different sugarcane species. To confirm the transcriptome data from RNA-Seq, 26 DEGs were selected randomly for qRT-PCR analysis (Figure 6). The qRT-PCR results were similar to the gene expression profiles in the transcriptome data, suggesting the transcriptome results were reliable.

### Association of Metabolic Analysis and Transcriptomic Analysis

To minimize the false positives single-omics analysis, the integrated analysis of KEGG pathway enrichment, functional analysis and correlation analyses were performed between the transcriptome and metabolome. All the DEMs and DEGs were mapped to the KEGG pathway database to identify the main biological pathways to better understand the relationship between genes and metabolites. KEGG enrichment analysis showed that pathways enriched in at least one omics data were 5, 15, 29, and 27 KEGG pathways (*p*-value < 0.05) in the 165204S_vs_FN41S, 165204L_vs_FN41L, 165204S_vs_165204L, and FN41S_vs_FN41L pair-wise comparison groups, respectively. The DEGs and DEMs were mainly enriched in phenylalanine metabolism, flavonoid biosynthesis, flavone and flavonol biosynthesis, starch and sucrose metabolism, glycine, serine and threonine metabolism, carbon metabolism, and citrate cycle (TCA cycle). Later, four pathways including phenylpropanoid biosynthesis (ko00940), flavonoid biosynthesis (ko00941), flavone and flavonol biosynthesis (ko00944), and starch and sucrose metabolism (ko00500) were selected for subsequent analysis to explore the potential links between the metabolome and the transcriptome data.

**FIGURE 7:**
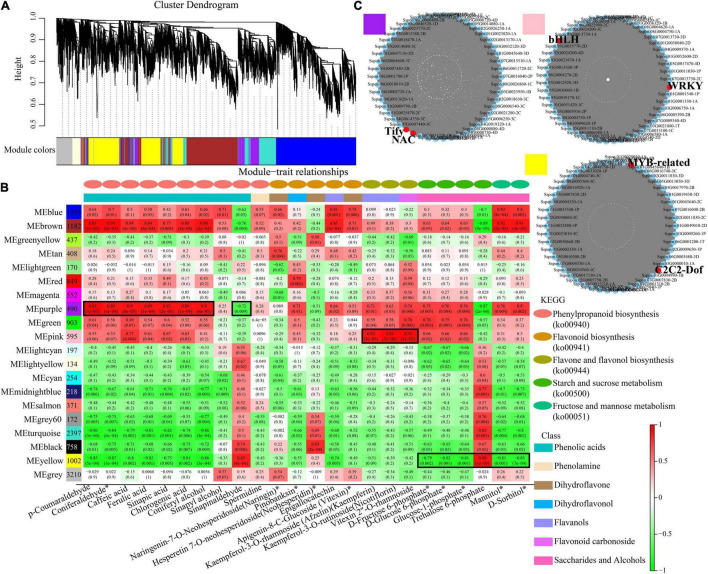
Co-expression network analysis. **(A)** Hierarchical cluster tree showing 20 modules obtained by weighted gene co-expression network analysis (WGCNA). The gray modules represent genes that are not divided into specific modules. Each branch in the tree points to a gene. **(B)** Matrix of module-metabolite associations. Combining the gene expression profile data of stem and leaf tissues of different sugarcane varieties and the change patterns of phenylpropanoid biosynthesis, flavone and flavonol biosynthesis, starch and sucrose metabolism, displayed by the WGCNA analysis. The number of genes in each module is shown in the left box, followed by correlation coefficient and *p*-value between modules and metabolites, which are displayed at the intersection of rows and columns. **(C)** Co-expression sub-network analysis of purple, pink, and yellow modules related to the accumulation of phenylpropanoid biosynthesis, flavone and flavonol biosynthesis, starch and sucrose metabolism. The first 50 nodes of purple, pink, and yellow modules to build the network were selected, and transcription factors are shown in red.

To further identify modules related to phenylpropanoid biosynthesis (ko00940), flavonoid biosynthesis (ko00941), flavone and flavonol biosynthesis (ko00944), and starch and sucrose metabolism (ko00500), the significantly changed phenolic acids, flavonoids and saccharides were combined with RNA-seq data to construct a co-expression network (Figure 7A and Supplementary Table 7). Twenty modules (labeled in different colors) were identified in the dendrogram, where the gray module represents genes that were not assigned to specific modules. Remarkably, the purple module showed a significant correlation with the accumulation pattern of phenylpropanoid biosynthesis (*r* > 0.85 or *r* < –0.85, *p* < 0.001), while the pink module showed a significant correlation with the accumulation pattern of flavone and flavonol biosynthesis (*r* > 0.9 or *r* < –0.9, *p* < 0.001). Whereas the yellow module showed a significant correlation with the accumulation pattern of starch and sucrose metabolism (*r* > 0.89 or *r* < –0.79, *p* < 0.001) (Figure 7B). Among these genes, 490 genes of the purple module were positively related to *p*-coumaraldehyde, sinapic acid, caffeic acid and coniferyl alcohol. We also noticed that 1,002 genes of the yellow module were negatively related to D-fructose-6P, D-glucose-6p, and α-D-glucose-1P. 595 genes of the pink module were positively related to kaempferin, nicotiflorin, and vitexin 2”-*O*-rhamnoside.

Based on the number of connections between genes in the co-expression network, the top 50 node genes in the purple, pink and yellow modules were selected to generate the co-expression subnetwork (Figure 7C and Supplementary Table 7). Among these hub genes, we found six transcription factors in the three modules, namely, Tify (Sspon.05G0031500-1C) and NAC (Sspon.06G0028920-1C), in the purple module, followed MYB-related (Sspon.01G0014260-1T) and C2C2-Dof (Sspon.04G0023660-4P), in the yellow module, and WRKY (Sspon.03G0003750-3C) and bHLH (Sspon.06G0010740-1A), in the pink module. These transcription factors play a key role in phenylpropanoid biosynthesis, flavone and flavonol biosynthesis, and starch and sucrose metabolism.

### Integrating Related Genes and Metabolites in the Phenylpropanoid and Flavonoid Biosynthesis Pathway

**FIGURE 8:**
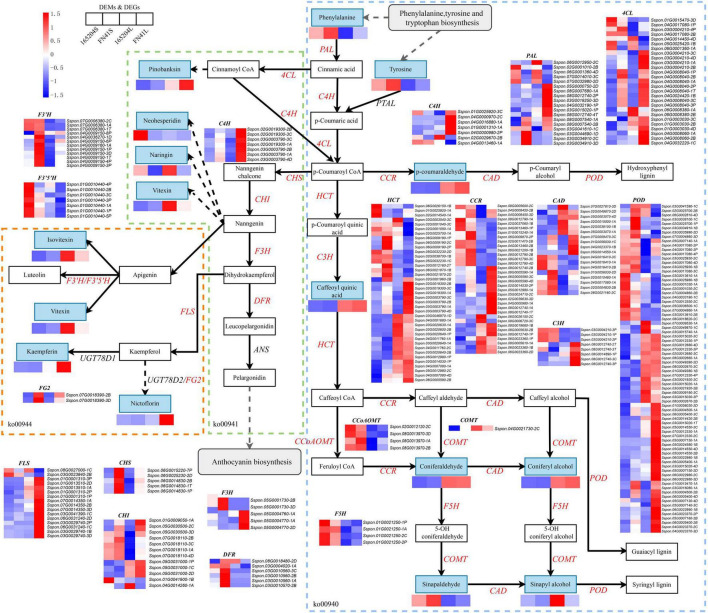
Diagram of phenylpropanoid and flavonoid biosynthesis pathways with their related DEGs and DEMs. PAL, phenylalanine ammonia lyase; PTAL, phenylalanine/tyrosine ammonia-lyase; C4H, cinnamate4-hydroxylase; 4CL, 4-coumarate CoA ligase; HCT, hydroxycinnamoyl CoA shikimate/quinate hydroxycinnamoyl transferase; C3H, P-coumarate 3-hydroxylase; COMT, caffeic acid *O*-methyltransferase; CCoAOMT, caffeoyl-CoA *O*-methyltransferase; CHI, chalcone isomerase; CCR, cinnamoyl-CoA reductase; CAD, cinnamyl alcohol dehydrogenase; F5H, ferulate5-hydroxylase; F3′5′H, flavonoid 3′,5′-hydroxylase; POD, peroxidase; FLS, flavonol synthase; DFR, dihydroflavonol 4-reductase; Non-significant DEGs are shown in black. The solid line indicates the metabolic reactions in only one step. The dash line presents more than one step of the metabolic reaction.

To elucidate the differences in the production of phenylpropanoids and flavonoids metabolism between the two sugarcane species, we identified and mapped the DEGs and DEMs that were predicted to be involved in the phenylpropanoid and flavonoid biosynthesis (Figure 8). We observed that a total of 16 DEMs were mapped to these pathways, including eight phenylpropanoid biosynthesis, four flavonoid biosynthesis four flavone and flavonol biosynthesis. The profiles of DEMs between the two tissues showed the content of *p*-coumaraldehyde, caffeoyl quinic acid, coniferaldehyde, coniferyl alcohol, sinapyl alcohol, pinobanksin, naringin, vitexin, nicotiflorin, kaempferin, and vitexin were more evident in the leaves than the stems. While the accumulation of L-phenylalanine, tyrosine, sinapinaldehyde, and neohesperidin in the stems were higher than that in the leaves. We also compared the DEMs between the two cultivars, it was observed that the precursors of the phenylpropanoid biosynthesis pathway (L-phenylalanine and Tyrosine) were more abundant in FN41S and FN41L than that in 165204S and 165204L, while sinapaldehyde, pinobanksin, kaempferin, and nictoflorin followed the same trend. The expression of pathway genes was also affected across different tissues. The majority of DEGs were observed to be both up-regulated and down-regulated, such as PAL, 4CL, HCT, CCR, CAD, C3H, POD, and CHI. However, some DEGs exhibited unique expression profiles in a specific species or tissue (specific expression data is shown in Supplementary Table 8).

**FIGURE 9:**
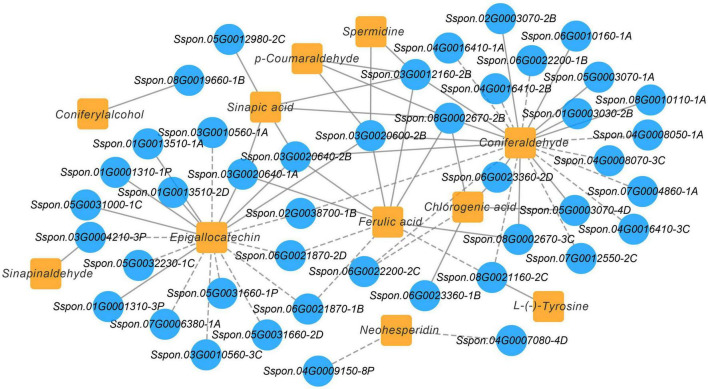
Co-expression analysis illustrating genes and metabolites in phenylpropanoid and flavonoid biosynthesis pathway. Nodes represent genes or metabolites, and edges represent relationships between any two genes. Edges with solid and dashed lines represent positive and negative correlations, respectively, as determined by a Pearson correlation coefficient > 0.8 or < –0.8, respectively.

A Pearson’s correlation coefficients (PCCs) analysis was performed to measure the degree of correlation between DEGs and DEMs. In the phenylpropanoid and flavonoid biosynthesis pathways, the results of the PCC calculation showed that forty-three DEGs were significantly associated with eleven DEMs, with the vast majority demonstrating positive association. Specifically, 43 pairs were significantly and positively correlated (PCC value > 0.8), whereas 27 pairs revealed significant and negative correlations (PCC value < –0.8). Among them, the number of differential genes associated with the coniferaldehyde were more (23 DEGs), followed by epigallocatechin (18 DEGs) (Figure 9). Besides, Sspon.03G0012160-2B was associated with the metabolites, namely, coniferaldehyde and spermidine, and demonstrated a highly positive correlation, followed by sinapic acid, *p*-Coumaraldehyde and chlorogenic acid, exhibiting a significant and positive correlation with Sspon.08G0002670-2B. Whereas Sspon.01G0001310-3P and Sspon.03G0020600-2B were significantly and positively correlated with epigallocatechin and ferulic acid, respectively.

### Integrating Related Genes and Metabolites in the Starch and Sucrose Metabolism

**FIGURE 10:**
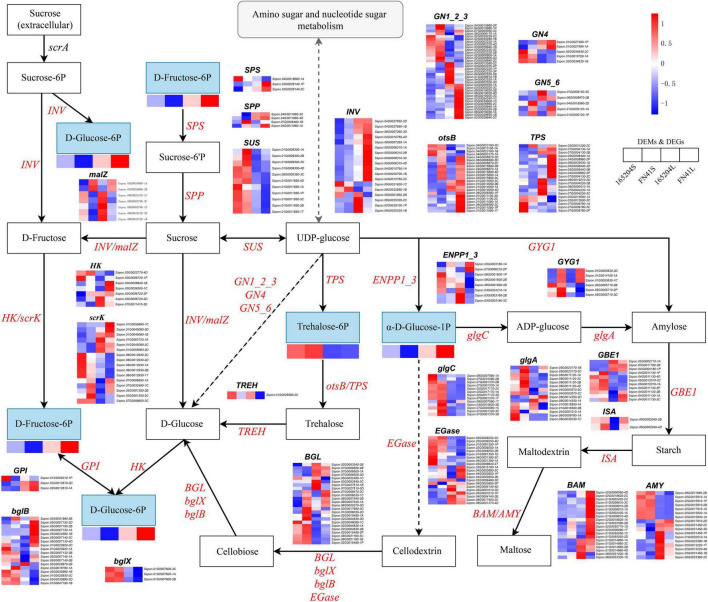
Diagram of starch and sucrose metabolism pathway with their related DEGs and DEMs. SPS, sucrose phosphate synthase; SPS, sucrose phosphatase; SUS, sucrose synthase; INV, invertase; HK, hexokinase; scrK, phosphofructokinase; non-significant DEGs are shown in black. The solid line indicates the metabolic reactions in only one step. The dash line presents more than one step of the metabolic reaction.

The DEGs and DEMs of starch and sucrose metabolism in the FN41 and 165204 of the two tissues were investigated using heat maps (Figure 10). Four DEMs were mapped including D-Fructose-6p, D-Glucose-6p, α-D-Glucose-1p and trehalose-6p. D-Fructose-6p, D-Glucose-6p and α-D-Glucose-1p were more pronounced in the leaves than the stems, while trehalose-6p exhibited the contrary. Interestingly, the content of D-fructose-6p, D-glucose-6p, and α-D-glucose-1p were more pronounced in FN41L and FN41S, respectively. Furthermore, we identified 315 DEGs classified into 26 gene families with diverse regulation patterns, highlighting the complex regulation of sugar metabolism in sugarcane (specific expression data is shown in Supplementary Table 9). We identified eighteen INV DEGs, the majority of which were significantly up-regulated in FN41 as compared to 165204 cultivar. Besides, two malZ genes (Sspon.03G0016860-1A&-2B) were found up-regulated in FN41, while the other five malZ genes (Sspon.08G0003790-3C&-1P, Sspon.08G0018720-1B&-2D, and Sspon.08G0025470-1C) were significantly down-regulated in FN41 In this study, we identified 11 SuS DEGs, all were up-regulated in the stems of the two cultivars. Among them, four SuS genes (Sspon.01G0009300-1A&-2B&-3C and Sspon.01G0011850-4D) were up-regulated in FN41 as compared to 165204, while the remaining SuS genes were down-regulated in FN41, indicating that SuS was negatively correlated with sugar accumulation. Three SPS DEGs and four SPP DEGs were also identified, of which two SPS (Sspon.03G0028140-2C&-1P) and two SPP (Sspon.07G0026460-1B and Sspon.04G0011960-1A) were upregulated in FN41, which indicate a higher sucrose formation in FN41.

It is worth noting that the results of PCC analysis between genes and metabolites showed that in the starch and sucrose metabolism pathway, trehalose 6-phosphate was the only metabolite significantly associated with 47 DEGs, of which 31 pairs were significantly and positively correlated, while 16 pairs were significantly and negatively correlated (Supplementary Figure 5). Among them, Sspon.02G0017170-2C, Sspon.02G0015810-2B, and Sspon.02G0017910-1A showed a significant and positive correlation with trehalose 6-phosphate.

## Discussion

Metabolomics is an effective tool for measuring metabolite composition of various plant tissues. Targeted and untargeted metabolomics techniques have also been used to identify and quantify metabolites present in different organs of plant species during different development stages (Wang et al., 2018; Xiao et al., 2021). In this study, a targeted metabolomic approach was adopted to investigate the metabolic changes in sugarcane stems and leaves of two contrasting cultivars, FN41 and 165204. A total of 512 metabolites from 11 classes were detected in the sugarcane stems and leaves. This finding was confirmed with the study conducted by Glassop et al. (2007) in which they identified 121 and 71 metabolites in cultivar TMS and TBS using metabolomics tool, respectively. Studies have revealed that flavonoids play a vital role in plant tissues color formation, plant development and food quality (Xu et al., 2015). Flavonoids are also the largest and the most studied group of plant phenols with variable phenolic structures which could be further divided into flavones, isoflavones, flavonols, flavanols, flavanones, and anthocyanins (Panche et al., 2016). For instance, Yang et al. (2022) revealed that flavonoids in vegetative tissues of rice were observed to be abundant. Correspondingly, we found that flavonoids were the most dominant metabolites in the two sugarcane cultivars, exhibiting distinct distribution patterns in the various plant tissues. KEGG pathway enrichment analysis further showed that a large number of metabolites, namely, phenylpropanoid biosynthesis, flavonoid biosynthesis, and flavone and flavonol biosynthesis pathways were enriched in the DEMs. This finding is in consonance with a previous study, wherein it documented that metabolites such as phospholipids, amino acids and most lipids and fatty acids were enriched during rice seed germination (Yang et al., 2022), suggesting that flavonoids play an important role in distinguishing the two phenotypes of sugarcane.

Sucrose synthesis takes place in the cytoplasm of the leaf pulp of sugarcane, and the main rate-limiting enzyme is SPS. SPS catalyzes the irreversible conversion of uridine diphosphate glucose (UDPG) to fructose-6P to form sucrose-6’p which has been immediately catalyzed by SPP to form sucrose. SPS is also known to be the key enzyme in resynthesizing sucrose from the hexoses in the sink tissue (Huber and Huber, 1996). In this study, we noticed that two SPS genes (Sspon.03G0028140-2C&-1P) and two SPP (Sspon.07G0026460-1B and Sspon.04G0011960-1A) that were up-regulated in FN41, which agreed with the finding reported by Nguyen-Quoc et al. (1999), wherein the overexpression of SPS gene in tomatoes results in increased sucrose loading and transport rate. Our result is also in line with the study conducted by Park et al. (2008). The authors mentioned that over-expression of an Arabidopsis SPS gene resulted in a considerable improvement of sink sucrose concentrations in tobacco (*Nicotiana tabacum* cv. Xanthi) plants. We therefore, postulated that the differences in sugar content among two sugarcane cultivars may largely be associated with the up-regulation of these genes in FN41.

Previous study has shown that the sugar content of the watermelon fruit is mainly determined by three enzyme families, sucrose synthase (SUS), SPS and insoluble acid convertase (IAI) (Liu et al., 2013). SUS can catalyze both sucrose synthesis and sucrose catabolism, but mainly convert UDP-glucose into sucrose (Schmölzer et al., 2016). The overexpression of a potato sucrose synthase gene in cotton enhances fiber production and sucrose supply by expanding the plant leaves (Xu et al., 2012). In tomato fruit, SUS contributes to the accumulation of glucose and fructose (Li et al., 2021). Nevertheless, in the present study 7 of the 11 SuS genes were down-regulated in FN41 and SuS was negatively correlated with sugar accumulation. This is probably due to the dual role of SUS, which also known to perform the catabolic function during sugarcane growth and development.

In plants, sucrose is irreversibly hydrolyzed by invertase (INV) into glucose and fructose (Koch, 2004). INV can be classified into cell-wall invertase (CWIN), vacuolar invertase (VIN), and cytoplasmic invertase (CIN) according to the cell location (Wan et al., 2018). CWIN catalyzes cytoplasmic sucrose hydrolysis, which is involved in sucrose cytoplasmic unloading and hexose supply for development; VIN has an important function in hexose accumulation, cell expansion; and CIN contains cytoplasmic sugar homeostasis. In a previous research, 14 INV members were cloned in sugarcane, and VIN was induced by fructose treatment (Wang et al., 2017). In the present study, the majority of INV was upregulated in FN41 and four INV genes (Sspon.02G0025100-1P, Sspon.05G0001850-2B, and Sspon.06G0025320-1B&-2C) were downregulated in FN41. This may be due to the fact that different INVs play versatile roles in sugar metabolism and signaling in sugarcane. Hexokinase (HK) is a fructose and glucose phosphorylating enzyme, and also act as a sugar sensor that may regulate sugar-dependent gene repression or activation (Jang et al., 1997). HK catalyzes the first committed step of glucose metabolism by converting glucose to D-glucose-6p (Dai et al., 1995). The biosynthesis of trehalose is accomplished through trehalose 6-phosphate synthase (TPS) and trehalose 6-phosphate phosphatase (otsB), and trehalose plays a protective role against stress in plants (Paul, 2007). Metabolites including D-fructose-6P, D-glucose-6p, α-D-glucose-1P, and trehalose-6P were involved in the starch and sucrose metabolism of sugarcane. The distributions of metabolites suggested that FN41 synthesizes more monosaccharides in photosynthetic organs (source tissues) to convert these into other forms of carbohydrates and transport them for storage in heterotrophic cells (sink tissues). Therefore, we inferred the metabolites in starch and sucrose metabolism could be important, and the manipulation of these metabolites-related genes could provide prospects for increasing sugar content in sugarcane.

The phenylpropanoid pathway not only gives rise to flavonoids, but also converts them into lignin and various other aromatic metabolites such as coumarins, phenolic volatiles, or hydrolyzable tannins (Vogt, 2010). Phenylalanine and tyrosine are aromatic amino acids (AAAs) that are used for the synthesis of proteins. In plants, high carbon flux is committed to the biosynthesis of phenylalanine and tyrosine because they serve as precursors of numerous natural products, such as pigments, alkaloids, hormones, and cell wall components (Maeda and Dudareva, 2012). We noticed that phenylalanine and tyrosine were significantly up-regulated in FN41, implying that FN41 has more metabolic substrates for subsequent metabolic synthesis and may produce more energy in the sugarcane stems. Lignin is one of the most important secondary metabolites and one of the main components of the plant cell wall that play an important role in plant development such as enhancing the overall mechanical strength of plants, promoting transportation through the vascular bundles (Boerjan et al., 2003). Phenylpropanoids such as sinapyl alcohol, coniferyl alcohol, and coumaryl alcohol act as important precursors of lignin biosynthesis (Liu et al., 2018). In apples, it was shown that the reduced levels of sinapaldehyde and *p*-coumaryl alcohol ultimately led to significant lignin loss and growth retardation (Zhou et al., 2019). Recent studies have also shown that the cellulose content decreased while lignin content increased during pigmentation of winter jujube, and guaiacyl-syringyl (G-S) lignin was the main lignin type in the pericarp (Zhang et al., 2021). A precursor of *S*-lignin and sinapaldehyde, was found to be significantly up-regulated in the expression of FN41 stems in this study, which we believed had effects on the color and fiber composition of the stems, thereby promoting the synthesis of *S*-lignin. Cinnamate 4-hydroxylase (C4H), a cytochrome P450-dependent monooxygenase, catalyzes the first oxidative step of the phenylpropanoid pathway in higher plants by transforming *trans*-cinnamate into *p*-coumarate, which is a key substrate required for the formation of all flavonoids (Ayabe and Akashi, 2006). Plant growth and lignin accumulation were inhibited in the Arabidopsis C4H mutant (Schilmiller et al., 2009).

A number of studies have revealed that the leaf and stem of sugarcane are have strong relationship with source and sink (Roopendra et al., 2018). In this study, the leaf area, flavonoid index and chlorophyll index in FN41L were more pronounced than that of 165204L, which is the key to the significant difference of sugar accumulation in stems of FN41 and 165204. We also observed that the sugar content of FN41S was significantly higher than that of 165204S. This finding is in agreement with the study conducted by Roopendra et al. (2018), wherein it was revealed that the sucrose content of cane culm, possibly influenced by source–sink variation in sugarcane tissue. We believed that the relatively high amount of D-fructose 6-p, D-glucose6-p, and glucose1-p detected in FN41L may have been transported and distributed by source and sink of the plant, and a majority of them reached the stem of sugarcane FN41L, thereby promoting the high accumulation of sugar in FN41L.

Differential gene analysis provides us with a correlation of the possible gene functions at developmental stages based on the changes in the expression levels of DEGs. However, each gene in the differential analysis is isolated, whereas, in reality, genes and gene products are composed of regulatory networks to perform functions. WGCNA is a widely used systems biology method for describing the correlation patterns among genes across different samples that could be used to effectively screen specific modules of interest with highly related genes (Langfelder and Horvath, 2008). In this study, three modules related to phenylpropanoid biosynthesis (ko00940), flavonoid biosynthesis (ko00941), flavone and flavonol biosynthesis (ko00944), and starch and sucrose metabolism (ko00500) were identified, including hub genes and six transcription factors. In a previous study, C2C2-Dof zinc finger family were found differentially expressed between immature and mature tissues in the high-fiber sugarcane only. They were also influenced cellulose and lignin metabolism as well as the prominent players in carbon metabolism (Lakshmi et al., 2018). In maize, *ZmDOF36* acted as a critical regulatory factor in starch synthesis, and could help devise strategies for modulating starch production in maize endosperm (Wu et al., 2019). The abnormal expression of *bHLH3* disrupts the balance of the network and redirects flavonoid metabolic flux in pale-colored fruits, resulting in differences in the levels and proportions of anthocyanins, flavones, and flavonols among differently colored mulberry fruits (Li et al., 2020). NAC domain-containing protein could be involved in many biological processes such as secondary wall biosynthesis and abiotic stress response (Olsen et al., 2005).

The color and texture of sugarcane stems are not only important quality indicators but also the critical parameters that affect the consumer acceptance of fresh sugarcane products. Moreover, flavonoids are not only the main compounds that determine the color of flowers, fruits and leaves but also play an important role in plant growth, development, and plant adaptation to environment. Flavonoid metabolites and their associated genes in several plants have been comprehensively studied (Albert et al., 2014). Many species of plants start to change color after the activation of the flavonoid-related enzymes (Vogt, 2010). Vitexin, isovitexin, and pinobanksin are active components of many medicinal plants and have received increased attention as their wide range of pharmacological effects, such as antioxidant, antivirus, and antibacterial effects (He et al., 2016). The detection of these metabolites in sugarcane in the present study demonstrated the feasibility of extracting antioxidant substances from sugarcane leaves and stems. Naringin and neohesperidin have been reported to be responsible for the bitterness of citrus and are mainly influenced by its sugar content (Wang et al., 2016). In this study, the contents of naringin and neohesperidin were significantly downregulated in FN41, implying that these two metabolites may have played significantly contributed to the sweet taste of sugarcane. We also detected four anthocyanins in this study, while metabolites did not differ significantly between the samples. This may account for that the sugarcane stem rind and stem pith being sampled in a mixed sample, resulting in a non-significant difference in anthocyanin content. Chalcone synthase (CHS) was the first enzyme to be identified in flavonoid biosynthesis and located at the upstream point of the flavonoid biosynthesis pathway (Kreuzaler and Hahlbrock, 1972). Previous studies revealed that expression of MdCHS3 from apple in poplar resulted in reduced total lignin content and increased cell wall carbohydrate content in transgenic poplar cell walls (Mahon et al., 2021). Furthermore, silencing of the CHS gene could shift the anthocyanin pathway to the synthesis of chlorogenic acid and its complexes, and CHS is a key regulatory protein for anthocyanin biosynthesis in red and nectarine peaches (Rahim et al., 2014). In this study, all of the CHS genes were significantly up-regulated in FN41, demonstrating that CHS may play an important role in the synthesis of anthocyanin and lignin of two sugarcane cultivars. Flavanone 3-hydroxylase (F3H) converts naringen into dihydrokaempferol, and Dihydroflavonol-4-reductase (DFR) reduces dihydrokaempferol to leucoanthocyanidin, followed by oxidation of colorless leucoanthocyanidin to the precursor of anthocyanidins catalyzed by anthocyanin synthase (ANS) (Springob et al., 2003). These three enzymes are key enzymes in the synthesis of flavonol and anthocyanin. It was demonstrated in orange carnation that pigments synthesis is restricted when F3H expression was inhibited (Zuker et al., 2002). In grape berries, sugar-induced anthocyanin accumulation and F3H expression (Zheng et al., 2009). The purple leaf trait of ornamental kale was controlled by a gene BoPr encoding a DFR (Liu et al., 2017). Flavonoid 3′,5′ hydroxylases (F3′5′H) and (F3′H) are required for the biosynthesis of flavones, flavanones, flavonols, and anthocyanins, and has the potential to determine the pattern of flavonoid B-ring hydroxylation (Ayabe and Akashi, 2006). Our study revealed that F3′H and F3′5′H were upregulated in FN41 and 165402, respectively, triggering competition for substrates between F3′5′H and F3′H. In the biosynthesis of flavonoids and flavanols, the UGT78D family catalyzes glycosylation and occurs at the O-3 or O-7 position (Kc et al., 2018). We observed that flavonol-3-*O*-glucoside L-rhamnosyltransferase (FG2) was upregulated in FN41.

## Conclusion

To conclude, we explored the molecular mechanism of differential sugar accumulation, rind color, and texture in two sugarcane cultivars. High sugar content was observed in FN41 as compared to 165204. Comparison of the differences in the level of metabolites and gene expression was performed. The analysis identified the metabolites and genes that have the potential to regulate sugar content, rind color, and texture in sugarcane. The results also suggested that genes such as C4H, CHS, F3H, F3′H, DFR, and FG2 in phenylpropanoid and flavonoid biosynthesis pathways may be a major factor impacting the rind color and contrasting texture of FN41 and 165204 sugarcane stems. Moreover, metabolites including L-phenylalanine, tyrosine, sinapaldehyde, pinobanksin, kaempferin, and nictoflorin were the potential drivers of phenotypic differences. Our findings also indicated that genes and metabolites in the starch and sucrose metabolism may have an important effect on sugar content in sugarcane. Overall, this study revealed molecular mechanisms underpinning the accumulation of sugar content, rind color, and texture of two sugarcane verities, which we believed is important for future sugarcane breeding programs and the selection of high biomass varieties. Up-regulated genes in FN41, namely, F3H, DFR, F3’H, and FG2 should be addressed in future studies to probe the specific mechanism.

## Data Availability Statement

The datasets presented in this study can be found in online repositories. The raw RNA-seq read data were deposited in the Short Read Archive (http://www.ncbi.nlm.nih.gov/sra/) and can be accessed using the BioProject ID: PRJNA805530.

## Author Contributions

All authors contributed to intellectual input and provided assistance to this study and manuscript preparation. ZY and ZP designed the research and conducted the experiments. FD analyzed the data and wrote the manuscript. YZ designed the qPCR primer and made RT-qPCR experiments. ZL, NF, and CH reviewed the manuscript. ZY supervised the work and approved the manuscript for publication.

## Conflict of Interest

The authors declare that the research was conducted in the absence of any commercial or financial relationships that could be construed as a potential conflict of interest.

## Publisher’s Note

All claims expressed in this article are solely those of the authors and do not necessarily represent those of their affiliated organizations, or those of the publisher, the editors and the reviewers. Any product that may be evaluated in this article, or claim that may be made by its manufacturer, is not guaranteed or endorsed by the publisher.

## Footnotes

1
https://www.shimadzu.com.cn/

2
http://www.massbank.jp/

3
http://kanaya.naist.jp/KNApSAcK/

4
http://www.hmdb.ca/

5
http://metlin.scripps.edu/index.php

6
www.r-project.org/

7
http://www.ncbi.nlm.nih.gov/sra/

8
https://www.genome.jp/kegg

9
http://itak.feilab.net/cgi-bin/itak/index.cgi

10
http://www.openbioinformatics.org/annovar/

11
https://www.ncbi.nlm.nih.gov/tools/primer-blast/

## Supplementary Material

Supplementary Figure 1 Diagram of the method of testing in tensile strength perpendicular to grain of wood.

Supplementary Figure 2 Total ion chromatograms (TIC) under positive **(A)** and negative mode **(B)**.

Supplementary Figure 3 Hierarchical clustering analysis of all metabolites detected in this study. The abscissa indicates three biological replicates of FN41stems (FN41S1, FN41S2, and FN41S3), 165204 stems (165204S1, 165204S2, and 165204S3), FN41 leaves (FN41L1, FN41L2, and FN41L3), and 165204 leaves (165204L1, 165204L2, and 165204L3), and the ordinate indicates the metabolites detected in this study. The red segments indicate a relatively high content of metabolites, while the blue segments indicate a relatively low content of metabolites.

Supplementary Figure 4 Heat map depicting correlation between biological replicate.

Supplementary Figure 5 Co-expression analysis of genes and metabolites in starch and sucrose metabolism pathway. Nodes represent genes or metabolites, and edges represent relationships between any two genes. Edges with solid and dashed lines represent positive and negative correlations, respectively, as determined by a Pearson correlation coefficient > 0.8 or <–0.8, respectively.

## Abbreviations

PAL: phenylalanine ammonia lyase
PTAL: phenylalanine/tyrosine ammonia-lyase
C4H: cinnamate4-hydroxylase
4CL: 4-coumarate CoA ligase
HCT: hydroxycinnamoyl CoA shikimate/quinate hydroxycinnamoyl transferase
C3H: *P*-coumarate 3-hydroxylase
COMT: caffeic acid *O*-methyltransferase
CCoAOMT: caffeoyl-CoA *O*-methyltransferase
CHI: chalcone isomerase
CCR: cinnamoyl-CoA reductase
CAD: cinnamyl alcohol dehydrogenase
F5H: ferulate5-hydroxylase
F3’5’H: flavonoid 3’,5’-hydroxylase
POD: peroxidase
FLS: flavonol synthase
DFR: dihydroflavonol 4-reductase.
