# Supplementary figures and images for: Integrated Metabolomics and Transcriptome Analyses Unveil Pathways Involved in Sugar Content and Rind Color of Two Sugarcane Varieties
Source: Front Plant Sci. 2022 Jun 16;13:921536. doi: 10.3389/fpls.2022.921536 (PMC9244704; doi:10.3389/fpls.2022.921536)

A

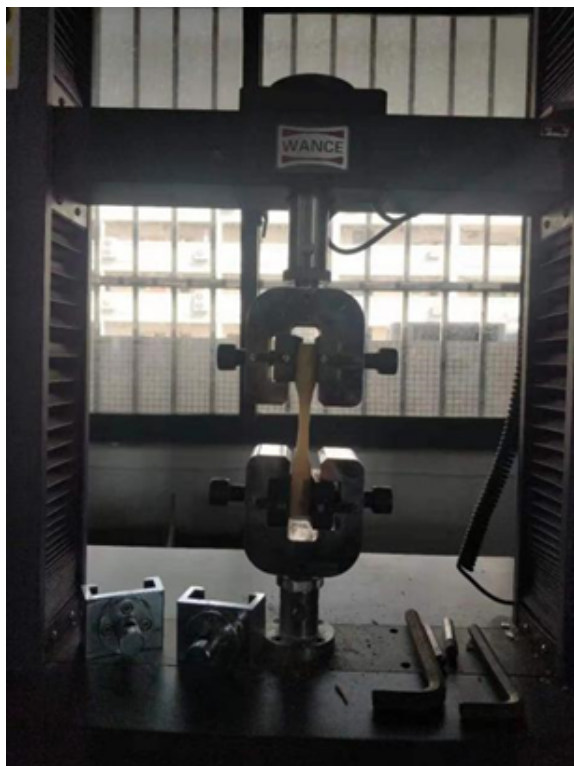

B

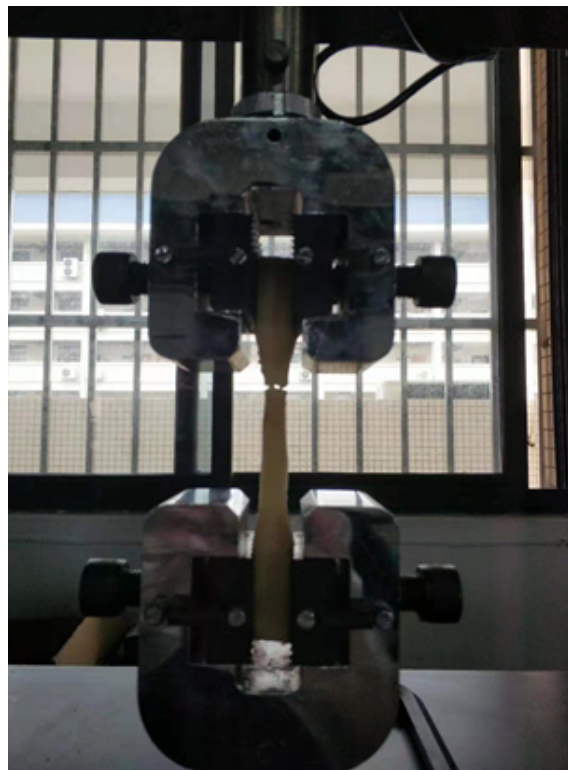

Supplement: Supplementary Figure 1 — Diagram of the method of testing in tensile strength perpendicular to grain of wood. [file Data_Sheet_1.PDF]

A

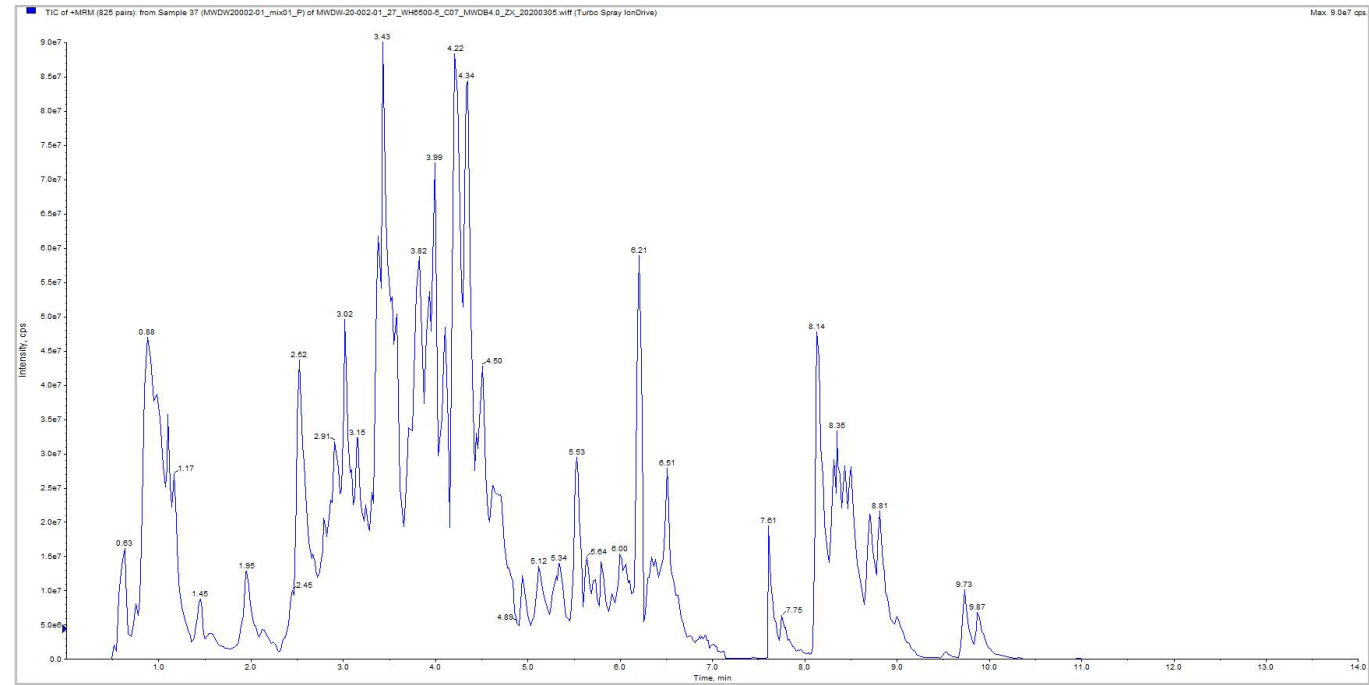

B

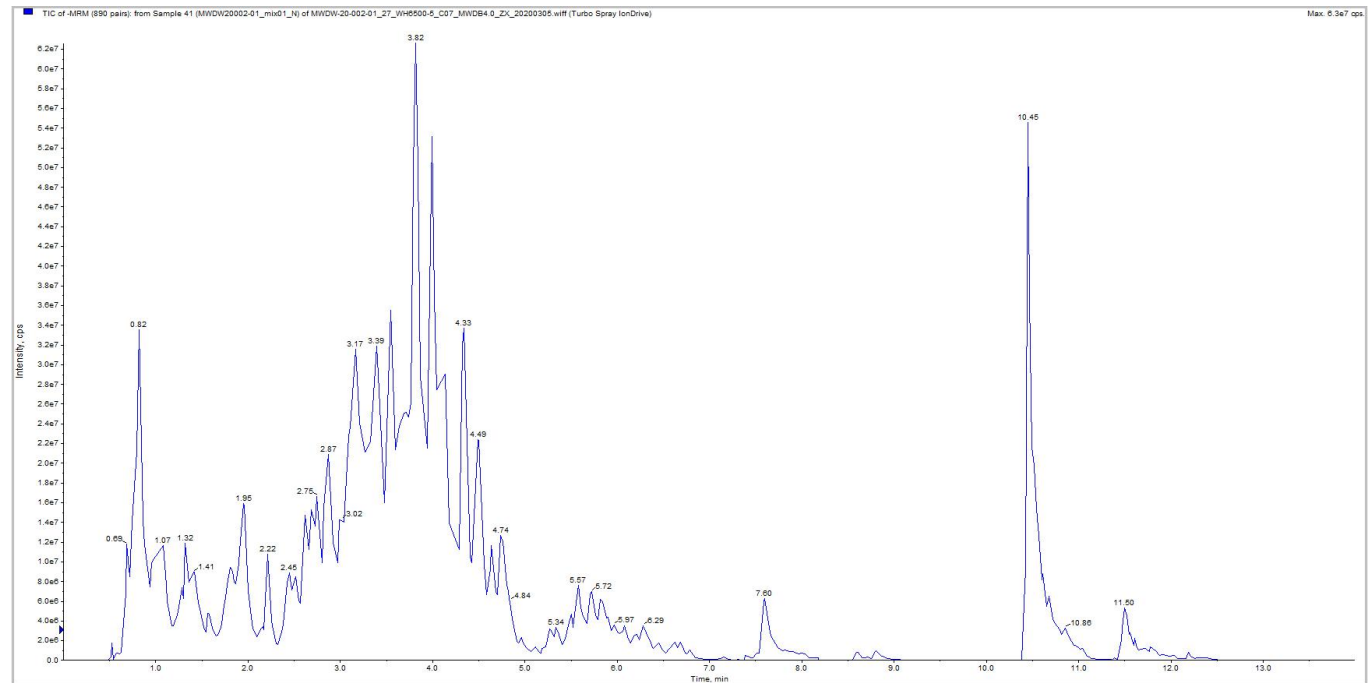

Supplement: Supplementary Figure 2 — Total ion chromatograms (TIC) under positive (A) and negative mode (B). [file Data_Sheet_2.PDF]

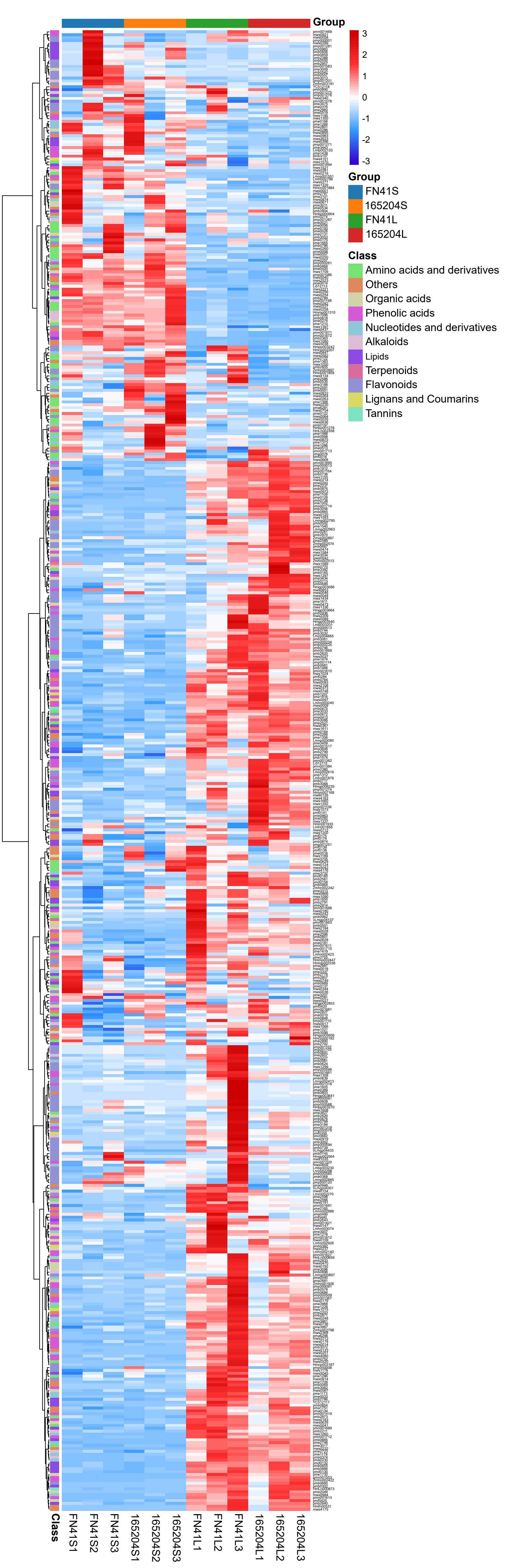

Supplement: Supplementary Figure 3 — Hierarchical clustering analysis of all metabolites detected in this study. The abscissa indicates three biological replicates of FN41stems (FN41S1, FN41S2, and FN41S3), 165204 stems (165204S1, 165204S2, and 165204S3), FN41 leaves (FN41L1, FN41L2, and FN41L3), and 165204 leaves (165204L1, 165204L2, and 165204L3), and the ordinate indicates the metabolites detected in this study. The red segments indicate a relatively high content of metabolites, while the blue segments indicate a relatively low content of metabolites. [file Data_Sheet_3.PDF]

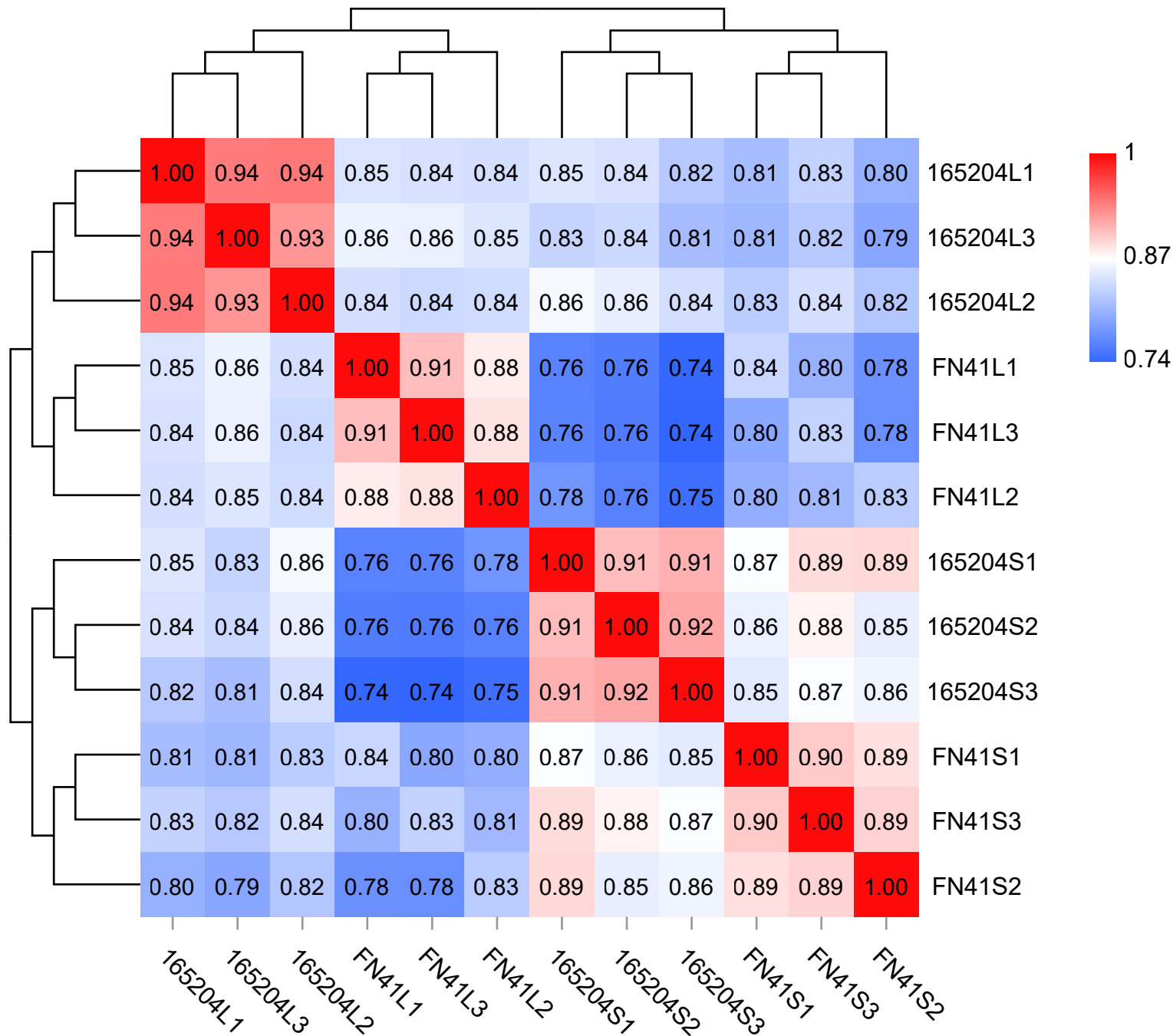

Supplement: Supplementary Figure 4 — Heat map depicting correlation between biological replicate. [file Data_Sheet_4.PDF]

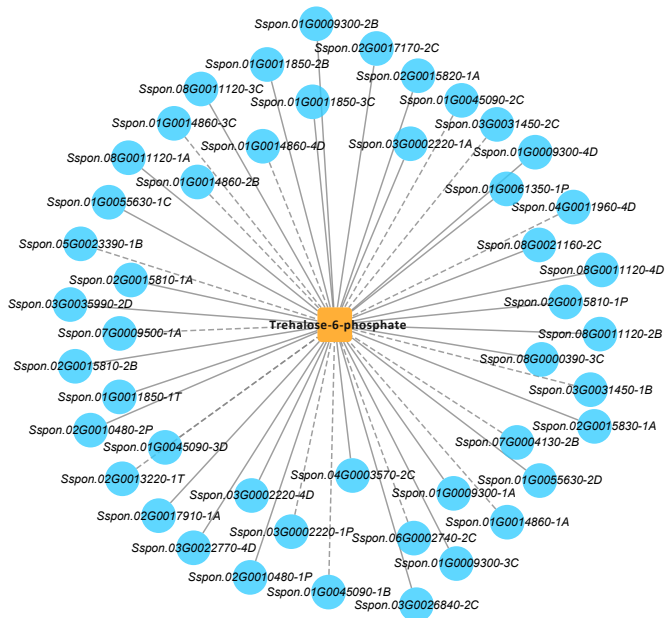

Supplement: Supplementary Figure 5 — Co-expression analysis of genes and metabolites in starch and sucrose metabolism pathway. Nodes represent genes or metabolites, and edges represent relationships between any two genes. Edges with solid and dashed lines represent positive and negative correlations, respectively, as determined by a Pearson correlation coefficient > 0.8 or <–0.8, respectively. [file Data_Sheet_5.PDF]
